# High frequency of benzimidazole resistance polymorphisms and age-class differences in trichostrongyle nematodes of ranched bison from the south-central United States

**DOI:** 10.1016/j.ijpddr.2025.100594

**Published:** 2025-04-14

**Authors:** Kaylee R. Kipp, Elizabeth M. Redman, Joe L. Luksovsky, Dani Claussen, John S. Gilleard, Guilherme G. Verocai

**Affiliations:** a Department of Veterinary Pathobiology, College of Veterinary Medicine and Biomedical Sciences, Texas A&M University, College Station, TX, USA; b Department of Comparative Biology and Experimental Medicine, Host-Parasite Interactions Program, Faculty of Veterinary Medicine, Hospital Drive, 3330, University of Calgary, Calgary, Alberta, Canada

**Keywords:** Anthelmintic drug resistance, *Haemonchus contortus*, Metabarcoding, North American bison, *Ostertagia ostertagi*, Trichostrongyloidea

## Abstract

Bison production is a growing sector of the United States agriculture, with more consumers choosing bison products. Commercial bison are kept on smaller pastures and treated with anthelmintics for gastrointestinal nematodes (GIN) to maintain production. However, there is a lack of information regarding the GIN parasite communities in ranched bison or the extent of their resistance to anthelmintics. Our objectives were: i) to determine the GIN species present and the extent of resistance to the benzimidazole drug class in commercial bison herds in the southern US and ii) to assess age class differences in GIN species composition and BZ resistance. Composite coprocultures from bison in Texas (*n* = 14) and Oklahoma (*n* = 2), and individual bison of different age classes from a single ranch in central Texas (*n* = 43) were analyzed using ITS2 rDNA nemabiome metabarcoding to determine the trichostrongylid species composition. For both the composite and individual samples, the most common trichostrongylid species found were *Haemonchus contortus, Haemonchus placei*, and *Ostertagia ostertagi*. Among the known canonical isotype-1 β-tubulin BZ resistance polymorphisms (at codons 200, 198, 167), the 200Y (TTC > TAC) substitution was the most widespread across the two southern states, with a prevalence of 81.3 %. Other polymorphisms, such as 167Y (TTC > TAC) and 198L (GAA > TTA), were also detected, and both had prevalences of 62.5 %. *Ostertagia ostertagi* was found to have very high frequencies (overall mean frequency = 62.6 %; range = 28.3–100 %) of the 200Y (TTC > TAC) polymorphism in all age classes sampled. Overall, benzimidazole resistance polymorphisms were found at moderate to high frequency in the three major economically important GIN species in ranched bison in Texas and Oklahoma, suggesting a potential widespread distribution of benzimidazole resistance polymorphisms in the southern United States. This work has important implications for all other grazing livestock and illustrates the importance of early detection of anthelmintic resistance and the need for mitigation strategies.

## Highlights

• We assessed trichostrongyle species composition in bison herds in south-central US.
• Markers for benzimidazole resistance in trichostrongyles found at high frequencies.
• Benzimidazole resistance is common in *H. contortus, H. placei*, and *O. ostertagi.*
• Age class differences were found in trichostrongyle species composition.

## Graphical abstract

**Figure:**
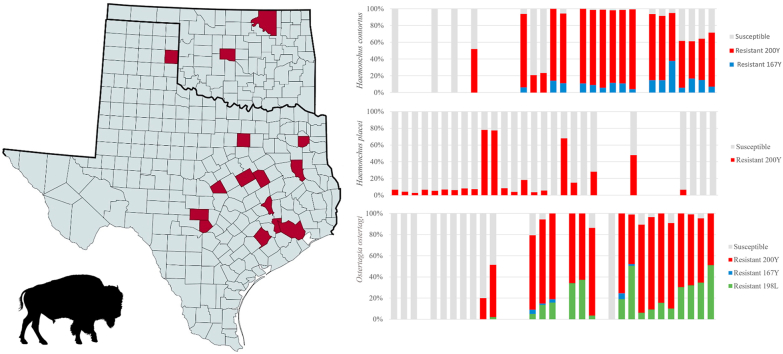

## 1 Introduction

The North American bison (*Bison bison*) is a keystone species across grassland ecosystems of North America (Knapp et al., 1999) and has become a unique and high-valued livestock species in the United States and Canada. The popularity of bison in both husbandry and consumption has led to more ranchers owning and raising bison for meat production to meet consumer demand (Bison Producers’ Handbook, 2015; National Bison Association, 2019). It is estimated that over 500,000 bison are farmed in North America, with a large majority owned and managed on private ranches (Bison Producers’ Handbook, 2015; National Bison Association, 2021). Though often thought of as being mainly found across the northern plains, many bison ranches are found in the southern United States, including Texas and Oklahoma. In fact, the 2022 Agriculture Census estimated a total of 8187 bison across 298 ranches in Texas and 7137 bison across 77 ranches in Oklahoma (USDA: Animal and Plant Health Inspection Service, 2019). Although many of these ranched bison are on smaller hobby farms, there are many large-scale production ranches whose goal is to produce marketable animals for consumer consumption. Despite not being domesticated, bison ranching has followed many of the same management strategies used in cattle production (Bison Producers’ Handbook, 2015; USDA: Animal and Plant Health Inspection Service, 2019). These bison can be raised in smaller, over-grazed, and over-stocked pastures leading to higher parasitic burdens (USDA: Animal and Plant Health Inspection Service, 2019). With this increase in parasitism throughout herds, producers have become more reliant on anthelmintics to control these production-limiting parasitic nematodes.

Gastrointestinal nematodes (GIN) within the order Strongylida often exist in complex communities within a host and can vary in their level of pathogenicity, drug sensitivity, and production effect (Avramenko et al., 2017; Charlier et al., 2022). These GIN, specifically those of the trichostrongylid group, are a major cause of disease and production loss in livestock species (Corwin, 1997; Fleming et al., 2006; Sutherland and Leathwick, 2011; Kaplan, 2020), including bison (Frick, 1951; Wade et al., 1979; Woodbury et al., 2014; Avramenko et al., 2018; Chelladurai et al., 2024). Anecdotal evidence and clinical cases suggest that bison herds often report GIN parasite infection intensities which can lead to more significant clinical signs and production loss compared to cattle (Frick, 1951; Wade et al., 1979; Woodbury et al., 2014; Avramenko et al., 2018). Though parasitism of GIN often causes subclinical disease, clinical disease, and mortality has been reported in ranched bison, causing greater economic losses within the industry (Frick, 1951; Wade et al., 1979; Tessaro, 1989; Eljaki et al., 2016; Avramenko et al., 2018).

Unfortunately, the control of GIN in grazing livestock can be challenging as regions of the country experience different obstacles based on climate, geographical distributions, and management strategies. Regional parasite diversity differences are evident in cattle throughout North America and are associated with climatic conditions (Stromberg et al., 2015; Hildreth and McKenzie, 2020; Navarre, 2020; De Seram et al., 2022). While information is available on parasites of bison in central and northern US (Frick, 1951; Locker, 1953; Boddicker and Hugghins, 1969; Wade et al., 1979; Eljaki et al., 2016; Chelladurai et al., 2024) as well as Canada (Tessaro, 1989; Dies and Coupland, 2001; Woodbury et al., 2014; Avramenko et al., 2018), knowledge on bison parasites across the southern United States is largely unstudied. However, in the southern states, warmer climates create favorable conditions for GIN transmission, thus, parasite transmission seasons are often longer in the southern states compared to northern states and Canada (Williams and Loyacano, 2001; Hildreth and McKenzie, 2020; Navarre, 2020). These climatic differences necessitate different parasite management strategies, including anthelmintic administration timing and different refugia management approaches (Morgan and Van Dijk, 2012; Hildreth and McKenzie, 2020; Navarre, 2020).

Studies from Canada and the northern United States show that bison share most of the common trichostrongylid GIN with cattle and small ruminants including species within the *Haemonchus, Ostertagia, Cooperia*, and *Trichostrongylus* genera (Tessaro, 1989; Van Vureni and Scott, 1995; Marley et al., 1995; Dies and Coupland, 2001; Woodbury et al., 2014; Eljaki et al., 2016; Avramenko et al., 2017). These trichostrongyle nematodes produce eggs that are morphologically indistinguishable making their identification virtually impossible at this stage. Feces can be cultured to allow trichostrongyle eggs to hatch and develop into their third-stage larvae (L3) allowing morphological identification to the genus level (Verocai et al., 2020; Van Wyk and Mayhew, 2013). However, this technique is very specialized, time-consuming, requires intensive training, and can often lead to false identification. These challenges have led to the development of a powerful molecular approach, referred to as “nemabiome” metabarcoding, which allows for species-level determination of GIN in individual samples through deep-amplicon sequencing of the ITS-2 nuclear ribosomal DNA locus (Avramenko et al., 2015).

Another largely understudied aspect is the level of anthelmintic resistance in the GIN in grazing ruminants, especially bison, in the southern United States. With all grazing ruminants harboring some level of parasitism, many livestock producers heavily rely on anthelmintics to control GIN burdens and maximize production. Overreliance and misuse of these drugs apply selective pressure, which is a potential risk factor for the emergence of anthelmintic resistance. While these impacts are relatively well-studied in cattle and small ruminants (Fleming et al., 2006; Sutherland and Leathwick, 2011; Gasbarre, 2014; Avramenko et al., 2019, 2020; Kaplan, 2020), information regarding the anthelmintic resistance in internal parasites in ranched bison is relatively scarce (Woodbury and Lewis, 2011; Avramenko et al., 2020). The emergence of anthelmintic resistance further impacts livestock production, causing economic losses to individual producers and the overall industry (Eljaki et al., 2016; Kaplan, 2020). Determining resistance is classically based on the fecal egg count reduction test, which can be difficult to perform when working with undomesticated animals. It is also an insensitive technique. Consequently, by the time resistance has been identified within a herd, it could be too late for mitigation strategies. Molecular testing has the ability to identify anthelmintic resistance early and with only one sampling time. Currently, the most well-characterized anthelmintic resistant mechanism is for the benzimidazole drug class (Gilleard, 2006; Avramenko et al., 2019; Kaplan, 2020). Avramenko et al. (2019) developed a high-throughput molecular assay to screen for polymorphisms (200Y [TTC > TAC], 167Y [TTC > TAC], 198L [GAA > TTA], and 198A [GAA > GCA]) in the isotype-1 β-tubulin gene that is associated with resistance to benzimidazoles in various GIN of sheep and later, cattle and bison (Avramenko et al., 2019, 2020). While resistance is relatively common and widespread in abomasal nematodes of cattle from the US (Gasbarre et al., 2009; Edmonds et al., 2010; Sutherland and Leathwick, 2011; Gasbarre, 2014; Avramenko et al., 2020), there are very few reports that specifically look at bison. When examining Canadian bison herds *Ostertagia ostertagi* benzimidazole resistance alleles were found at low frequency in a few herds (7/51) (Avramenko et al., 2020).

As well as anthelmintic resistance status, the overall parasite diversity remains to be explored in bison in the southern United States, where management practices, climate, and parasite species composition differ from the northern United States and Canada. In this paper, we apply nemabiome ITS2 metabarcoding and screen for benzimidazole resistance polymorphisms in the isotype-1 β-tubulin gene to investigate the species composition of parasite communities and the frequency level of benzimidazole resistance in composite bison herds in Texas and Oklahoma as well as individual bison of three different age classes (calves, yearlings, and mature) from a single ranch in east-central Texas. This is one of the first in-depth papers assessing the species composition and level of resistance in GIN of bison from the southern United States, and the first to assess bison parasites in Texas, using next-generation sequencing techniques.

## 2 Materials and methods

### 2.1 Collection of fecal samples

#### 2.1.1 Archival composite samples

**Fig. 1:**
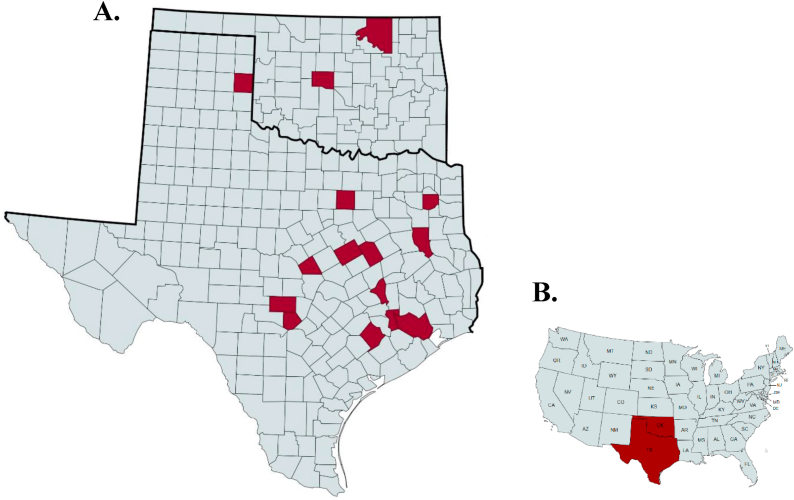
(A) Geographical distribution of commercial bison ranches in various counties, highlighted in red, in Texas and Oklahoma that sent in fecal samples. (B) Geographical location of Texas and Oklahoma in reference to the United States.

Samples were collected from bison herds in Texas (n = 14) and Oklahoma (n = 2) from 2018 to 2022 (Fig. 1). Many of the sampled herds encompass north and central Texas, characterized by a humid subtropical climate with mild winters and hot summers. Other areas of sample collection in Texas include the Panhandle, which is a semi-arid climate with hot summers and cold winters (ClimateDataTexas.org). The two samples from Oklahoma were from central and northeastern regions and are characterized as having a subtropical climate with mild winters with the northern regions having a colder winter compared to the south (ClimateDataOklahoma.org). The two Oklahoma herds and four of the Texas herds were sampled at multiple time points giving the total number of samples selected to be 11 and 22, respectively. Herd composites comprised an average of 10 individual bison in Texas (range = 5–21) and 16 in Oklahoma (range = 7–20). Samples were sent either in the Spring (March–May; 15.6 %), Summer (June–August; 28.1 %), Fall (September–November; 43.8 %), or Winter (December–February; 12.5 %). Individual fecal samples were collected either per rectum by producers in a chute or from the ground after visual confirmation and animal identification, placed in plastic bags devoid of air, and shipped on ice to the Texas A&M University Parasitology Diagnostic Laboratory for general routine diagnostic analysis. Because of the highly variable management practices among herds, it is not possible to give a full description of each. However, each herd was given a routine anthelmintic to maintain production. Fecal egg counts (FEC) were determined using a modified Wisconsin technique with Sheather's sucrose floatation solution (specific gravity: 1.26). For each FEC 5 g of feces were used to give a theoretical detection threshold of 0.2 eggs per gram (EPG) of feces. The remaining fecal samples for each herd were then pooled together by age class (e.g., young [<3 years], mature [>3 years]) and cultured for 14 days at room temperature (∼20 °C) to hatch and develop the trichostongyle nematodes. Larvae were then estimated and aliquoted into 1000 larvae per herd if possible, however in some cases fewer than 1000 larvae were collected (range = 200–1000). Larvae were fixed in 70 % ethanol and stored at −80 °C until further analysis.

#### 2.1.2 Samples to assess GIN species composition among different age classes

To assess GIN species composition differences associated with age class, individual fecal samples were rectally collected from bison (n = 43) from a ranch located in the Brazos Country, central Texas (30° 38′5″ N, 96°20′31″ W) in April of 2022. Bison samples were classified according to their reported age classes: calves (n = 14; <1 year), yearlings (n = 3; 1-2 year-old), and mature (n = 26; >3 years). This herd was selected due to the known over reliance of albendazole which has been administered twice each year for the last +5 years, in combination with an injectable macrocyclic lactone. It should be noted that this herd rotates pastures but does not co-graze with cattle. The bison rotationally graze on four pastures that average 40 acres and maintain a relatively low stocking rate. The continued use of albendazole within the herd is largely due to the known presence of infection by liver fluke, *Fasciola hepatica*. Fecal egg counts were determined using the Mini-FLOTAC technique with sodium nitrate solution (FECA-MED, VDCO; specific gravity = 1.25–1.30), and a detection level of 5 EPG (Cringoli et al., 2017; Paras et al., 2018). The remaining fecal samples were individually cultured for 14 days for the collection of trichostrongylid L3 using the same protocol described above. The number of L3 obtained from each individual coproculture was estimated and aliquoted into 1000 L3 per herd, if possible (range = 100–1000), fixed in 70 % ethanol, and stored at −80 °C until further analysis.

### 2.2 Molecular analysis

#### 2.2.1 First round PCR for ITS-2 rDNA nemabiome metabarcoding

The species composition of GIN in each herd from both Texas and Oklahoma were determined using ITS-2 nemabiome metabarcoding of L3 larval populations using the Illumina Miseq platform (Avramenko et al., 2015). For the preparation of genomic DNA, ethanol-fixed larvae were washed in lysis buffer (50 mM KCl, 10 mM Tris (pH 8.3), 2.5 mM MgCl_2_, 0.45 % Nonidet P-40, 0.45 % Tween 20, 0.01 % (w/v) gelatin) three times by centrifugation before using the QIAamp PowerFecal Pro DNA kit for DNA extraction. After DNA extraction, the ITS-2 rDNA amplicon was PCR-amplified using previously published primers that consisted of four forward (NC1Adp, NC1Adp1N, NC1adp2N, NC1Adp3N) and four reverse (NC2Adp, NC2Adp1N, NC2Adp2N, NC2Adp3N) primers in equal proportions (Avramenko et al., 2015). The high-fidelity polymerase KAPA HiFi HotStart (KAPA Biosystems, USA) was used to minimize PCR error rates. The PCR consisted of 5 μL x5 Buffer, 15.15 μL of ddH_2_O, 0.75 μL dNTPs (10 mM), 0.75 μL NC1+Adapter Primer (10 mM), 0.75 μL NC2+Adapter Primer (10 mM), 0.1 μL BSA, and 2 μL of the DNA template. The thermocycling parameters for the PCR were 95 °C for 2 min, followed by 35 cycles of 98 °C for 20 s, 62 °C for 15 s, 72 °C for 15 s, followed by the final extension of 72 °C for 2 min. Following amplification, PCR products were purified using AMPure XP magnetic beads (ratio 1:1; Beckman Coulter, Inc.), following the manufacturer's recommended protocol.

#### 2.2.2 First round PCR for Isotype-1 β-tubulin deep-amplicon sequencing

The same samples were also screened for the presence and frequency of three independent SNP in the isotype-1 β-tubulin gene that are associated with benzimidazole resistance, namely 200Y (TTC > TAC), 167Y (TTC > TAC), 198L (GAA > TTA), and 198A (GAA > GCA) (Avramenko et al., 2020). The isotype-1 β-tubulin gene was PCR-amplified using previously reported primer sets which included a mix of 7 forward primers and 11 reverse primers (Avramenko et al., 2019; Supplemental Table 1) and the Q5 HotStart polymerase (New England Biolabs). The PCR consisted of 5 μL x5 Buffer, 11.65 μL of ddH_2_O, 0.5 μL dNTPs (10 mM), 1.25 μL Forward Primers (10 μM), 0.75 μL Reverse Primers (10 μM), 0.25 μL Q5 HotStart Polymerase (0.5 U), 0.1 μL BSA, and 5 μL of the DNA template. The thermocycling conditions for the PCR were 98 °C for 2 min, followed by 40 cycles of 98 °C for 10 s, 60 °C for 15 s, 72 °C for 25 s followed by the final extension of 72 °C for 5 min. The PCR product was then purified with AMPure XP Magnetic Beads (ratio 1:1).

#### 2.2.3 Illumina library preparation

For both ITS2 rDNA and isotype-1 β-tubulin bead-purified amplicons, a second limited cycle PCR amplification was completed for the addition of Illumina indices and P5/P7 sequencing tags. All samples were barcoded with Illuminas' Unique Dual Indexes (UDI). This consists of the addition of 384 unique 12bp indexes to both flanks of the amplicon. The second PCR conditions were as follows: 5 μL KAPA HiFi HotStart Fidelity Buffer (5X), 1.25 μL Forward Primers (10 μM), 1.25 μL Reverse Primers (10 μM), 0.75 μL dNTPs (10 mM), 0.5 μL KAPA HiFi Polymerase (0.5 U), 8.75 μL H_2_O, and 5 μL of the first round PCR product as the template. The thermocycling conditions for the second PCR were 95 °C for 45 s followed by 11 cycles of 98 °C for 20 s, 63 °C for 20 s, and 72 °C for 2 min. Products were purified using AMPure XP Magnetic Beads (1X). Purified products were then pooled (∼50 ng) to create a normalized sequencing library and quantified with the KAPA qPCR Library Quantification Kit (KAPA Biosytems, USA) following the manufacturer's recommended protocol. An Illumina MiSeq Desktop Sequencer (Illumina Inc., San Diego, CA, USA) using a 500-cycle pair-end reagent kit (MiSeq Reagent Kits v2, MS-103-2003) with the addition of a 25 % PhiX Control v3 (Illumina, FC-110-3001) was used to sequence the prepared pooled library at a concentration of 12.5 nM. An average read depth of 11,144 ± 4107 (range = 4230–21,878) was obtained for each sample.

### 2.3 Bioinformatic and statistical analysis

#### 2.3.1 Nemabiome ITS-2 bioinformatics

A bioinformatics pipeline based on the analysis package DADA2 R version 1.14.0 (Callahan et al., 2016) was used to process the raw data (described in detail, https://www.nemabiome.ca). In brief, raw data is demultiplexed, barcode indices are removed and fastq files for each sample are generated. The pipeline uses the program Cutadapt version 2.8 (Marcel, 2011) to remove primers and filter the reads based on size (>200bp) and quality (utilizing the *filterAndTrim* function), discarding reads with a maximum of two expected errors in the forward read or five in the reverse read. Sequencing errors are removed using a dynamic error learning approach called “denoising” and the forward and reverse reads are then merged. Possible chimeric sequences are identified and removed from the dataset. The resulting Amplified Sequence Variants (ASV) are classified using IDTaxa (Murali et al., 2018) at a confidence threshold of 60 % against the nematode ITS2 rDNA database 1.1.0 (Workentine et al., 2020). The ASVs were processed further to remove singletons, potential contaminants, and samples with less than 1000 reads and then converted into species proportions based on the total number of reads per sample.

#### 2.3.2 Isotype-1 beta tubulin bioinformatics

A bioinformatics pipeline based on the analysis package DADA2 R version 1.14.0 (Callahan et al., 2016) was used to process the raw data in a similar approach as the ITS-2 pipeline; the reads were filtered, trimmed, denoised, and merged using default settings. The resulting ASVs were aligned against reference sequences (Avramenko et al., 2019) using a global (Needleman-Wunsch) pairwise alignment algorithm without end gap penalties. Following alignment, the ASVs were discarded if they were <180 bp or >350 bp long, or if they had a percentage identity <70 % to any of the reference sequences in the database. The ASV was also removed if it had fewer than 200 reads in a sample and if the ASV was only present in a single sample. Additionally, samples with <1000 reads per sample were excluded from further analysis. The codons 167, 198, and 200 were then analyzed for the presence of any variants resulting in non-synonymous changes. The frequency of each of the resistance polymorphisms for each species was estimated as a proportion of reads with the resistance polymorphisms out of the total number of mapped reads for a given species.

#### 2.3.3 Statistical analysis of variables

The resulting data from the nemabiome was statistically analyzed using the Statistical Analysis System (SAS; Version 9.4, SAS Institute, Cary, North Carolina, USA) to determine the prevalence for each GIN species and the three known benzimidazole resistance markers across herds. To assess the effect of age differences on FEC and sequence polymorphism frequencies a generalized linear mixed model (GLMM) was determined using the PROC GLIMMIX procedure in SAS. In this model age group, sex, year, and state were fixed effects, and individuals or herds were the random effect to account for repeated measures at multiple time points. Because EPG data is skewed, a gamma distribution with a log link function was specified within the model. Other descriptive statistics were also determined including mean, standard deviation, and standard error. Alpha diversity was calculated for each age class in the central Texas herd using the inverse Simpson index.

## 3 Results

### 3.1 Fecal egg counts of GIN in archival composite samples

**Fig. 2:**
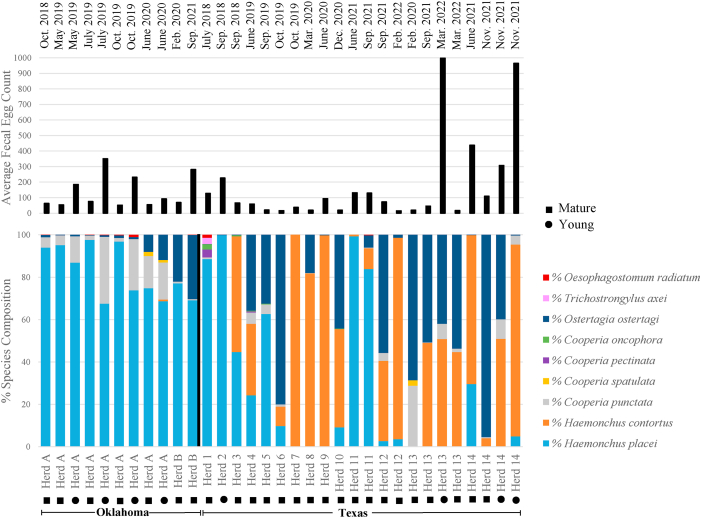
Infection intensities and species composition of parasitic gastrointestinal nematodes from commercial bison herds in Texas (n = 14) and Oklahoma (n = 2) are shown. Texas herds are shown as herds 1–14 and Oklahoma herds are depicted as A and B. Herds that repeat in number or letter are across multiple time points. The date of each time point is represented at the top of the figure. The upper portion of the chart shows the mean fecal egg counts (FEC) of trichostrongyles in each bison herd across Texas and Oklahoma. The lower portion of the chart shows the relative proportion of each parasite species present as determined by the nemabiome sequencing. Each color represents a different trichostrongyle as indicated by the legend. Age classes (Mature = >3 years; Young = <3 years) within herds are characterized by their affiliated shape at the bottom of the figure and are indicated in the legend.

Fecal egg counts were performed on individual animals submitted from each herd for diagnostic purposes. The overall EPG mean for trichostrongyles in Texas (n = 14) and Oklahoma (n = 2) herds was 179.1 ± 59.7 EPG (range = 17.1–997.7) and 137.4 ± 32.2 EPG (range = 54.4–350.5), respectively (Fig. 2). There were three herds analyzed (Texas = 2; Oklahoma = 1) that had submitted samples representing different age groups, including Herd A which had different age groups over multiple time points (Fig. 2). Considering these three herds overall we can observe the differences in EPGs between mature (>3 years; 93.1 ± 39.2 EPG) and younger bison (<3 years; 419.6 ± 125.6 EPG). Other helminths and protozoa were also recorded and summarized in Supplementary Table 2 by herd and state.

### 3.2 Species composition of GIN in archival composite samples

We used ITS-2 nemabiome metabarcoding, to determine the species composition of GIN for each herd composite in Texas and Oklahoma. The most predominant GIN species found in Texas overall were *Haemonchus contortus* (11/14 herds; mean (±SE) relative abundance = 42.2 ± 7.7 %), *Ostertagia ostertagi* (13/14 herds, mean relative abundance = 28.4 ± 6.4 %)*,* and *Haemonchus placei* (11/14 herds, mean relative abundance = 25.6 ± 7.8 %*;* Table 1, Fig. 2). Other GIN were also present, though in lower relative abundances, throughout Texas herds including four *Cooperia* species, *Trichostrongylus axei*, and *Oesophagostomum radiatum* (Table 1, Fig. 2). However, the distribution of these GIN species was variable between ranches, with some species detected only in one (*Cooperia spatulata* and *T. axei*) or two (*Cooperia pectinata* and *O. radiatum*) herds (Fig. 2). Despite *H. placei* being one of the most predominant GIN species found overall in Texas, higher levels (>60 %) were only found in 5 of the 16 herds, with most of the herds in Texas having lower proportions of *H. placei,* compared to *H. contortus* (Fig. 2).

**Table 1:** Average relative abundance of individual parasite species detected from ITS-2 rDNA nemabiome metabarcoding from Texas (14 herds) and Oklahoma (2 herds).

Species	Texas	Oklahoma
	Mean (%) ± SE	Mean (%) ± SE
*Haemonchus placei*	25.6 ± 7.8	82.0 ± 3.7
*Haemonchus contortus*	42.2 ± 7.7	0.1 ± 0.1
*Cooperia punctata*	3.1 ± 1.4	10.5 ± 3.2
*Cooperia spatulata*	0.1 ± 0.1	0.3 ± 0.2
*Cooperia pectinata*	0.2 ± 0.2	0.0 ± 0.0
*Cooperia oncophora*	0.2 ± 0.1	0.002 ± 0.002
*Ostertagia ostertagi*	28.4 ± 6.4	7.1 ± 3.1
*Trichostrongylus axei*	0.1 ± 0.1	0.0 ± 0.00
*Oesophagostomum radiatum*	0.1 ± 0.1	0.1 ± 0.1

In Oklahoma, *H. placei* (2/2 herds; mean (±SE) relative abundance = 82 ± 3.7 %) was the most abundant in both herds (A = 83.9 ± 4.2 %; B = 73.2 ± 4.0 %), time points, and age groups (herd A: yearling = 74.3 ± 4.4 %; mature = 91.7 ± 4.3 %) (Fig. 2). *Cooperia punctata* (2/2 herds; mean relative abundance = 10.5 ± 3.2 %) and *O. ostertagi* (2/2 herds; mean relative abundance = 7.1 ± 3.1 %) were also present, yet in much smaller proportions (Table 1). *Haemonchus contortus*, *C. spatulata*, *Cooperia oncophora*, and *O. radiatum* comprised an average of <1 % of the parasite community in Oklahoma herds (Table 1, Fig. 2). In fact, *H. contortus*, *C. spatulata*, and *C. oncophora* were only present in a single herd (i.e., herd A) (Fig. 2).

### 3.3 Benzimidazole resistance polymorphisms in GIN in archival composite samples

**Fig. 3:**
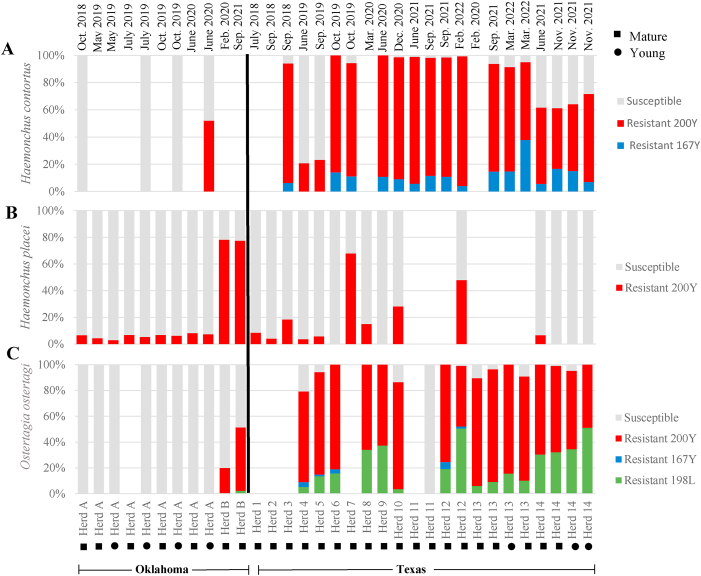
Benzimidazole resistance mutations in Texas and Oklahoma commercial bison herds. The allele frequency at codons 200, 198, and 167 of the β-tubulin isotype-1 gene is shown for *Haemonchus contortus* (A), *Haemonchus placei* (B), and *Ostertagia ostertagi* (C) derived from 16 composite herds samples from Texas (14) and Oklahoma (2) across multiple time points, as determined by deep-amplicon sequencing. Susceptible alleles are displayed in blue, while documented resistance alleles 200Y (TTC > TAC), 167Y (TTC > TAC), and 198L (GAA > TTA) are represented in the legend as red, blue, and green, respectively. Blank bars indicate that the species was either not present in the sample, or there were too few sequences (<200) assigned to the species to assess the allele frequency.

Benzimidazole resistance was determined by screening the trichostrongylid L3 for isotype-1 β-tubulin single nucleotide polymorphisms (SNPs) known to be associated with benzimidazole resistance at codons 200, 198, and 167 using deep amplicon sequencing. There was an overall high mean (±SE) frequency of the 200Y (TTC > TAC) polymorphism in *O*. *ostertagi* (mean frequency = 71.7 ± 3.1 %) and *H. contortus* (mean frequency = 70.6 ± 5.5 %) in almost every Texas bison herd, and low to moderate levels in *H*. *placei* (mean frequency = 20.6 ± 6.8 %) (Fig. 3; Table 2). The 167Y (TTC > TAC) polymorphism was also detected in *H. contortus* and *O. ostertagi*, with an overall mean frequency of 12.2 ± 2.0 % and 3.2 ± 0.8 %, respectively. Lastly, the 198L (GAA > TTA) polymorphism was only detected in *O*. *ostertagi* and had a mean frequency of 23.0 ± 3.9 % (Fig. 3; Table 2). Using the data available in the Texas herds, there was a significant difference (P = 0.007) in the overall 200Y (TTC > TAC) polymorphism frequency in *O. ostertagi* over the years (2019–2022) with a gradual increase (2019 = 36 %; 2020 = 70 %; 2021 = 60 %; 2022 = 83 %).

**Table 2:** 200Y (TTC > TAC), 167Y (TTC > TAC), and 198L (GAA > TTA) resistant polymorphism frequency in Texas and Oklahoma commercial bison herds.

	Polymorphism	Species	# Herds	# Herds SNP detected	Range (%)
			Species detected		
Texas (14 herds)	200Y	*Haemonchus contortus*	11	11	20.8–95.2
		*Haemonchus placei*	11	9	3.5–67.9
		*Ostertagia ostertagi*	13	9	46.8–87.3
	167Y	*Haemonchus contortus*	11	10	4.1–37.9
		*Haemonchus placei*	11	0	N/A
		*Ostertagia ostertagi*	13	4	1.4–5.6
	198L	*Haemonchus contortus*	11	0	N/A
		*Haemonchus placei*	11	0	N/A
		*Ostertagia ostertagi*	13	9	3.5–51.0
Oklahoma (2 herds)	200Y	*Haemonchus contortus*	1	1	52.0
		*Haemonchus placei*	2	2	2.85–78.2
		*Ostertagia ostertagi*	2	1	20.0–49.3
	167Y	*Haemonchus contortus*	1	0	N/A
		*Haemonchus placei*	2	0	N/A
		*Ostertagia ostertagi*	2	0	N/A
	198L	*Haemonchus contortus*	1	0	N/A
		*Haemonchus placei*	2	0	N/A
		*Ostertagia ostertagi*	2	1	2.1

The 200Y (TTC > TAC) resistance polymorphism was also the most predominant resistant polymorphism found in the Oklahoma samples, though at a low frequency. The 200Y (TTC > TAC) polymorphisms in *H. placei* was detected in both herds (A = 6.03 %; B = 77.9 %), across all time points (Fig. 3.), and in both age classes (herd A: yearlings [ylg] = 5.4 %; mature = 6.5 %). Though only found in low frequencies the frequency of the 200Y polymorphism, in both age classes in herd A, were observed to slowly increase over the months and years as follows: May 2019 (ylg = 2.9 %; mature = 4.2 %), July 2019 (ylg = 5.4 %; mature = 6.7 %), October 2019 (ylg = 6.2 %; mature = 6.8 %), and June 2020 (ylg = 7.3 %; mature = 8.2 %). *Ostertagia ostertagi* in herd B were found to have both the 200Y (TTC > TAC) (34.6 %) and 198L (GAA > TTA) (2.1 %) polymorphisms n, though the latter at a much lower frequency. Herd A was the only herd in Oklahoma that detected the 200Y (TTC > TAC) codon in *H. contortus* (52 %), which was found in high frequency only in yearlings from the last time point analyzed (June 2020) (Fig. 3).

### 3.4 Fecal egg counts of GIN in individual bison of different age classes

**Fig. 4:**
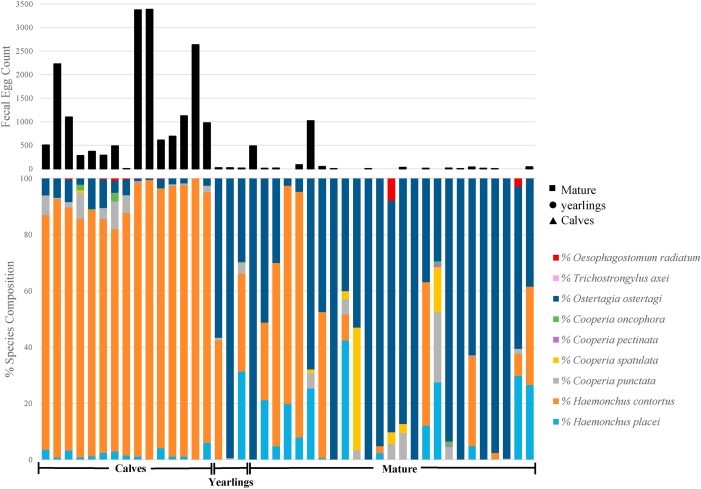
Infection intensities and species composition of parasitic gastrointestinal nematodes rectally collected from a single commercial bison herd in east-central Texas, along with three different age classes (calves, yearlings, and mature), are shown. Age classes within herds are characterized by their affiliated shape at the bottom of the figure and are indicated in the legend. The upper portion of the chart shows the mean fecal egg counts (FEC) of trichostrongyles in each individual bison within the central Texas ranch. The lower portion of the chart shows the relative proportion of each parasite species present as determined by the nemabiome sequencing. Each color represents a different trichostrongyle as indicated by the legend.

The average strongyle FEC in each age class was as follows; calves = 922.1 ± 162.5 EPG (14/14; range = 5–3385 EPG), yearlings = 340.0 ± 325.0 EPG (3/3; range = 20–975 EPG), and mature = 70.8 ± 110.4 EPG (16/26; range = 0–1020) (Fig. 4; S2. 3). There was an overall significant difference between the age groups with calves reporting higher EPG compared to mature bison (P = 0.0001). Other helminths and protozoa were also recorded in the Supplementary Table 3 by age class.

### 3.5 Assessing age class species composition of GIN in the single east-central Texas ranch

ITS-2 nemabiome metabarcoding was used to determine the species composition of GIN for individual animals in each age class (Fig. 4). There was a stark difference between the calves and mature bison (p < .0001) with *H. contortus* being more predominant in calves (mean frequency [±SE] = 90.4 ± 1.8 %), compared to yearlings (43.9 ± 25.8 %) and mature (18.6 ± 5.3 %) (Table 3). However, *O. ostertagi* was the predominant species in the mature bison (mean frequency = 65.7 ± 6.4 %) followed by yearlings (52.9 ± 28.0 %; calves = 4.5 ± 0.95 %), with a significant difference (p < .0001) between mature and calves (Fig. 4; Table 3). Other GIN species present within the herd were as follows, in order of overall prevalence: *H. placei* (6.7 %), *O. radiatum* (0.29 %), *C. punctata* (2.5 %), *C. spatulata* (1.7 %), *C. oncophora* (0.19 %), and *C. pectinata* (0.04 %) (Table 3). The alpha diversities for each age class were calculated using the inverse-Simpson index and were as follows: calves (0.18), yearling (0.53), mature (0.52).

**Table 3:** Average relative abundance of individual parasite species detected from ITS-2 rDNA nemabiome metabarcoding across different age classes from a single commercial bison herd from east-central Texas.

Species	Calves	Yearlings	Mature
	Mean (%) ± SE	Mean (%) ± SE	Mean (%) ± SE
*Haemonchus placei*	1.70 ± 0.35^a^	2.0 ± 2.0^a^	9.87 ± 2.59^a^
*Haemonchus contortus*	90.36 ± 1.83^a^	43.88 ± 25.75^ab^	18.61 ± 5.34^b^
*Cooperia punctata*	2.89 ± 0.99^a^	1.22 ± 0.54^a^	2.52 ± 1.03^a^
*Cooperia spatulata*	0.07 ± 0.07^a^	0.00 ± 0.00^a^	2.73 ± 1.75^a^
*Cooperia pectinata*	0.00 ± 0.00^a^	0.00 ± 0.00^a^	0.06 ± 0.05^a^
*Cooperia oncophora*	0.39 ± 0.25^a^	0.00 ± 0.00^a^	0.11 ± 0.07^a^
*Ostertagia ostertagi*	4.46 ± 0.95^a^	52.90 ± 28.03^ab^	65.69 ± 6.35^b^
*Oesophagostomum radiatum*	0.13 ± 0.06^a^	0.00 ± 0.00^a^	0.41 ± 0.32^a^

^a,b^ within a row, means without a common superscript differ (*P* < .0001).

### 3.6 Benzimidazole resistance polymorphisms in GIN in the single east-central Texas ranch

**Fig. 5:**
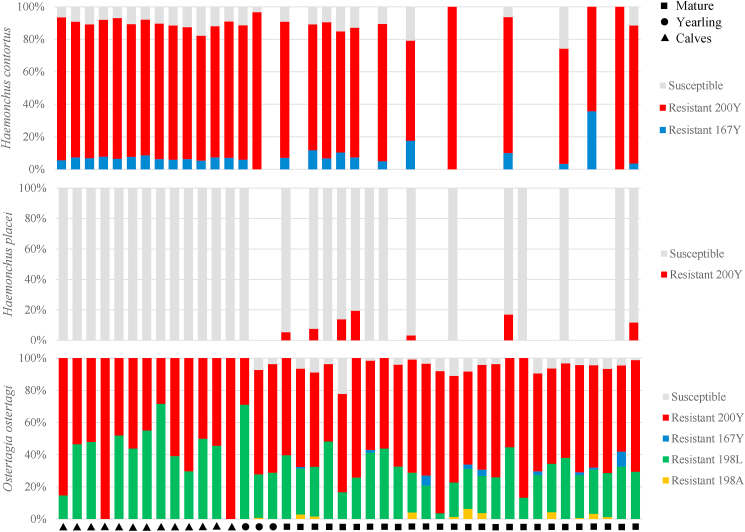
Benzimidazole resistance mutations in individual bison separated by age class (Calves, Yearlings, and Mature) from a single east-central Texas commercial bison ranch. The allele frequency at codons 200, 198, and 167 of the β-tubulin isotype-1 gene is shown for *Haemonchus contortus* (A), *Haemonchus placei* (B), and *Ostertagia ostertagi* (C) as determined by deep-amplicon sequencing. Susceptible alleles are displayed in blue, while documented resistance alleles 200Y (TTC > TAC), 167Y (TTC > TAC), 198L (GAA > TTA), and 198A (GAA > GCA) are represented in the legend as red, blue, green, and orange respectively. Blank bars indicate that the species was either not present in the sample, or there were too few sequences (<200) assigned to the species to assess the allele frequency.

Benzimidazole resistance polymorphisms in the isotype-1 β-tubulin gene at codons 200, 167, and 198 were screened using deep amplicon sequencing. There was a very high mean frequency of the 200Y (TTC > TAC) resistance polymorphism in *H. contortus* (59.3 %) in all age groups (calves = 83 %; yearling = 59.8 %, mature = 40.4 %) and *O. ostertagi* (44.4 %; calves = 61.9 %; yearlings = 53.8 %; mature = 64.1 %) (Fig. 5; Table 4). The 200Y (TTC > TAC) resistance polymorphism was also detected in low frequency in *H. placei but* was only detected in mature bison (3.01 %) (Fig. 5; Table 4). The second most predominant allele was the 198L (GAA > TTA) resistance polymorphism which, though was only detected in *O. ostertagi*, had a moderately high mean frequency of 32.7 % (calves = 38.15 %; yearlings = 42.3 %; mature = 28.8 %), compared to the 198A allele (1 %), which was also found in both 1-2 year-olds (0.3 %) and mature (1.1 %) but in low frequencies (Fig. 5; Table 4). Lastly, the 167Y (TTC > TAC) resistance polymorphism was detected in *H. contortus* (5.1 %) in all three age classes (calves = 6.8 %; yearlings = 2 %; mature = 4.6 %), while 167Y (TTC > TAC) in *O. ostertagi* was only detected mature bison (1.2 %) (Fig. 5; Table 4).

**Table 4:** 200Y (TTC > TAC), 167Y (TTC > TAC), 198L (GAA > TTA), 198A (GAA > GCA) resistant mutation frequency in three age classes from a single commercial bion herd from east-central Texas.

	Polymorphism	Species	# Herds	# Herds SNP detected	Range (%)
			Species detected		
Calves (n = 14)	200Y	*Haemonchus contortus*	14	13	76.9–88.0
		*Haemonchus placei*	12	0	N/A
		*Ostertagia ostertagi*	13	13	28.3–100
	167Y	*Haemonchus contortus*	14	13	5.3–7.9
		*Haemonchus placei*	12	0	N/A
		*Ostertagia ostertagi*	13	0	N/A
	198L	*Haemonchus contortus*	14	0	N/A
		*Haemonchus placei*	12	0	N/A
		*Ostertagia ostertagi*	13	11	14.6–71.7
	198A	*Haemonchus contortus*	14	0	N/A
		*Haemonchus placei*	12	0	N/A
		*Ostertagia ostertagi*	13	0	N/A
Yearlings (n = 3)	200Y	*Haemonchus contortus*	2	2	82.7–96.8
		*Haemonchus placei*	1	0	N/A
		*Ostertagia ostertagi*	3	3	28.9–64.9
	167Y	*Haemonchus contortus*	2	1	6.0
		*Haemonchus placei*	1	0	N/A
		*Ostertagia ostertagi*	3	0	N/A
	198L	*Haemonchus contortus*	2	0	N/A
		*Haemonchus placei*	1	0	N/A
		*Ostertagia ostertagi*	3	3	27.0–71.1
	198A	*Haemonchus contortus*	2	0	N/A
		*Haemonchus placei*	1	0	N/A
		*Ostertagia ostertagi*	3	1	0.8
Mature (n = 26)	200Y	*Haemonchus contortus*	14	13	61.5–100
		*Haemonchus placei*	13	7	3.3–19.5
		*Ostertagia ostertagi*	26	26	48.1–88.4
	167Y	*Haemonchus contortus*	14	11	3.3–37.1
		*Haemonchus placei*	13	0	N/A
		*Ostertagia ostertagi*	26	9	0.9–9.3
	198L	*Haemonchus contortus*	14	0	N/A
		*Haemonchus placei*	13	0	N/A
		*Ostertagia ostertagi*	26	26	3.6–48.2
	198A	*Haemonchus contortus*	14	0	N/A
		*Haemonchus placei*	13	0	N/A
		*Ostertagia ostertagi*	26	10	0.6–6.3

## 4 Discussion

Using metabarcoding and deep amplicon sequencing approaches, we were able to look at “snapshots” of 16 different ranched bison herds from two southern states in the United States, Texas and Oklahoma, from 2018 to 2021 to determine the species composition and screen for benzimidazole resistance polymorphisms. We investigated the species diversity and benzimidazole resistance in cohorts of individuals over different time points and, in some cases, between age classes for a subset of herds. Management strategies between the different ranches vary greatly with little to no prior knowledge of anthelmintic history. However, the common use of albendazole as an oral drench as well as the ease of giving fenbendazole range cubes, suggested there would be potentially a high selection pressure for benzimidazole resistance in many of the herds.

All nine GIN species found in the sampled bison are primarily those commonly associated with cattle and/or small ruminants. Our findings are similar to previous studies in ranched bison from Canada, and the northern United States (Avramenko et al., 2018; Eljaki et al., 2016; Avramenko et al., 2020). Nemabiome metabarcoding found the predominant GIN parasite species in Texas herds to be *H. contortus, H. placei,* and *O. ostertagi*. The higher prevalence and relative abundance of *H. contortus,* a parasite most often associated with small ruminants, in Texas bison contrast with what has been previously reported in other bison herds where *H. placei,* associated with cattle, was predominant (Avramenko et al., 2018, 2020). However, Texas is number one in the United States for the total number of sheep and goats produced (SheepProduction-2024.pdf; Cooperative Extension, 2019). Though producers of sheep and goats are often localized in the Edwards Plateau region of west-central Texas, there are many small ruminant herds throughout Texas. The two Oklahoma samples, including every time point in herd A, were *H. placei* with few *Cooperia* species. and *O. ostertagi*. A much lower number of herds were sampled in Oklahoma (n = 2) than in Texas (n = 14) and should be acknowledged that associations between states were not the goal of this current study, rather we aimed to report descriptive findings of herds that have never been reported previously. Although it was not unexpected to find *Haemonchus* spp. and *O. ostertagi* in southern regions of the United States, like Texas and Oklahoma, many herds sampled had high percentages of these production-limiting parasites. Previous studies conducted in the northern United States and Canada found *Cooperia* spp. to be the most abundant trichostrongylid nematode overall in bison herds, followed by either *Trichostrongylus* (Dies and Coupland, 2001) or *O. ostertagi* (Avramenko et al., 2018). However, *Haemonchus* spp. is known to be more adaptable to warmer weather and is often found in higher prevalence in subtropical and temperate climates, like found in the southern United States, compared to the northern United States and Canada (Terrill et al., 2012; Gasser and von Samson-Himmelstjerna, 2016; Charlier et al., 2022). These findings are of importance due to the pathogenicity of both *Haemonchus* spp. and *O. ostertagi* in all grazing ruminants (Terrill et al., 2012), including bison (Cameron, 1923; Frick, 1951; Wade et al., 1979; Tessaro, 1989).

Selection pressure on GIN from anthelmintic drug use will be a risk factor for the emergence of anthelmintic resistance in any livestock species, including bison. We screened each compositely sampled bison herd for the presence of different benzimidazole drug resistance polymorphisms in the isotype-1 β-tubulin gene in 200Y (TTC > TAC), 167Y (TTC > TAC), 198A (GAA > GCA), and 198L (GAA > TTA). Each of these polymorphisms has been previously identified in one or more trichostrongylid nematodes in small ruminants (Redman et al., 2015; Chagas et al., 2016; Avramenko et al., 2019), cattle (Chaudhry et al., 2014; Avramenko et al., 2020), and bison (Avramenko et al., 2020). However, resistance levels in ranched bison in the southern United States have never been investigated. Avramenko et al. (2020) did look at cattle herds in the central and southern United States, which included cattle from Oklahoma. The 200Y (TTC > TAC) polymorphism was detected in *H. placei* (frequency range = 0.57–27.45 %) and *O. ostertagi* (frequency range = 0.29–12.03 %)*,* which is similar to what was found in the present study, where the 200Y (TTC > TAC) substitution was the most prevalent in *H. contortus* (frequency range = 5.78–95.24 %)*, H. placei* (frequency range = 2.85–78.18 %)*,* and *O. ostertagi* (frequency range = 20.02–87.33 %), both in Texas and Oklahoma. Although this is consistent with previous studies, resistance levels were much lower in frequency overall (15.2 %) between herds (Avramenko et al., 2020). However, for the herds in Texas, there were high frequencies of the 200Y (TTC > TAC) polymorphism in *H. contortus* (mean frequency [±SE] 57.7 ± 7.5 %) and *O. ostertagi* (mean frequency 52.1 ± 7.3 %). Detecting the 200Y (TTC > TAC) polymorphism in such high levels in highly pathogenic and economically important parasites should be of great concern to not just the bison industry, but all grazing production animals. The frequency levels in the polymorphisms 198L (GAA > TTA) and 167Y (TTC > TAC) were considerably lower than the 200Y (TTC > TAC) polymorphism in herds from Oklahoma, as seen in Fig. 3. The 167Y (TTC > TAC) codon in *H. contortus* was the only other polymorphism detected. However, in *O. ostertagi*, codons 198L (GAA > TTA) and 167Y (TTC > TAC) were also found, but at a lower frequency. Despite their relatively lower frequency levels, 198L (GAA > TTA) was found in more than half of the bison herds in Texas. This is similar to what has previously been reported where the 200Y (TTC > TAC) polymorphism is more predominant compared to 198L (GAA > TTA), 198A (GAA > GCA), and 167 (TTC > TAC) in various livestock parasite species (Garg and Yadav, 2009; Gilleard, 2006, Avramenko et al., 2019; Avramenko et al., 2020).

Using the same molecular methods described previously for the composite archival samples from Texas and Oklahoma, we also investigated species composition and the presence of BZ resistance polymorphisms in GIN of individual bison within a single herd in east-central Texas. This provided the ability to look at individuals in more detail within three different age classes co-grazing in the same pastures. Though there was a lack of yearling data due to the limited sampling, there was a striking difference between the calves and the older bison with *H. contortus* being significantly higher in the younger animals (p < .0001) but *O. ostertagi* predominating in the older animals (p < .0001) (Fig. 2; Table 3). Though this is the first paper to look at the GIN species composition in different age classes in bison herds, others have observed age-related differences in cattle. It has been reported that calves often will have a higher abundance of *Haemonchus* spp., and as they mature *O. ostertagi* will become more prominent. This is thought to be due to age-related acquired immunity and species competition (Charlier et al., 2022). This age-related species difference is of great importance due to the high pathogenesis of *H. contortus* in young animals. Bison calves are not often separated at weaning; rather, they remain with the herd their whole life. However, calves that are continually grazing in pastures with mature bison increase their exposure to pathogenic GIN species within the herd. Coupled with higher resistance in these parasites, this can prove to be problematic for calves battling high parasitic burdens with little reduction after treatment, compared to mature bison that often experience lower parasitic burdens compared to calves. The east-central Texas ranch sampled for assessing age-class differences was of particular interest due to some management practices implemented. Specifically, the usage of albendazole which has been administered twice each year for the last +5 years, in combination with an injectable macrocyclic lactone. The continued use of albendazole within the herd is largely due to the known presence of infection by liver fluke, *Fasciola hepatica*. Consequently, we hypothesized that higher frequencies of benzimidazole resistance polymorphisms would be found, and this was supported by the detection of very high frequencies of benzimidazole resistance polymorphisms in both *H. contortus* and *O. ostertagi* across all age classes in this herd. Indeed, when considering the polymorphisms at codons 167, 198, and 200 combined in this population, the overall frequency of benzimidazole resistance polymorphisms for *O. ostertagi* was 94.48 % (frequency range = 48.2–100 %) in this herd. The finding of four different resistance polymorphisms 200Y (TTC > TAC), 167Y (TTC > TAC), 198A (GAA > GCA), and 198L (GAA > TTA), at such a high combined frequency is striking and suggests a high level of drug selection has occurred in this herd resulting in effectively every *O. ostertagi* worm in the population having a benzimidazole resistant genotype, This is the first paper to report such high levels of benzimidazole resistance polymorphisms in *O. ostertagi*. Polymorphisms associated with benzimidazole resistance have been reported in *O. ostertagi* before but generally at a much lower frequency (Avramenko et al., 2020). Knapp-Lawitzke et al. (2015) were able to create high frequencies of the β-tubulin codon 200 using experimental infections of calves. There have been previous reports of benzimidazole resistance in *H*. *contortus* and *H. placei*. Similar to what was found in the present study, Chaudhry et al. (2014), reported a low frequency of the β-tubulin codon 200 in *H. placei* in cattle in the southern United States. The high-frequency levels could be due to the over and misuse of anthelmintics in this bison herd. In addition, the bison screened rotate pastures with cattle which are also given anthelmintic treatment twice a year. Though it can be speculated, it is impossible to know who the selection was through originally, the overuse of anthelmintics could have started in the cattle herd and resistant GIN were subsequently picked up by the bison herds and is further fueled by their own continual dosage of anthelmintics over the years, leading to increased levels of resistance.

Further investigation should be made to determine how widespread benzimidazole resistance is in the central and southern United States, where favorable conditions for GIN occur most of the year. These findings have major implications for not just the bison industry but all susceptible grazing livestock. With increased animal movement, producers could unknowingly be purchasing bison harboring resistant GIN onto their property, potentially infecting other grazing animals that rotate the pasture. This could have a major economic impact on livestock industries such as cattle, sheep, and goats, which rely on anthelmintics to maintain production.

## 5 Conclusion

The misuse and overuse of anthelmintics in grazing livestock have contributed to increased levels of anthelmintic resistance in GIN populations which are known to transmit cross-species. While the bison industry is relatively new, benzimidazole resistance polymorphisms have already been reported at low frequency, in several GIN species in commercial bison in Canada and different parts of the United States. This is the first paper to examine the species composition and benzimidazole resistance in bison herds in the southern United States as well as to investigate age-related differences in individual bison using next-generation deep-amplicon sequencing. Our findings show that not only are SNPs at codons 167, 198, and 200 present in bison herds, but most appear to be widespread and present in high frequencies in economically important trichostrongyle nematodes, in special *O. ostertagi* and *H. contortus.* More specifically, our finding of such a high frequency of benzimidazole resistance in *O. ostertagi* is of great concern due to its effects on adult bison and minimal previous reports. This highlights the importance of early detection of resistance, proper stewardship of anthelmintics, and the implementation of mitigation strategies.

## CRediT authorship contribution statement

**Kaylee R. Kipp:** Writing – review & editing, Writing – original draft, Methodology, Investigation, Formal analysis, Data curation. **Elizabeth M. Redman:** Writing – review & editing, Methodology, Formal analysis, Data curation. **Joe L. Luksovsky:** Writing – review & editing, Methodology, Formal analysis, Data curation. **Dani Claussen:** Writing – review & editing, Methodology, Data curation. **John S. Gilleard:** Writing – review & editing, Supervision, Resources, Methodology, Investigation, Formal analysis. **Guilherme G. Verocai:** Writing – review & editing, Supervision, Resources, Project administration, Methodology, Funding acquisition, Conceptualization.

## Conflict of interest

The authors declare no conflict of interest.
