# Supplementary material for: High frequency of benzimidazole resistance polymorphisms and age-class differences in trichostrongyle nematodes of ranched bison from the south-central United States
Source: Int J Parasitol Drugs Drug Resist. 2025 Apr 14;28:100594. doi: 10.1016/j.ijpddr.2025.100594 (PMC12791206; doi:10.1016/j.ijpddr.2025.100594)
Supplement: Multimedia component 1 [file mmc1.docx]

**Supplementary Table 1.** Sets of primers used for screening the isotype 1 beta-tubulin gene of different trichostrongyle nematodes.

| **Orientation** | **primer name** | **primer** |
| --- | --- | --- |
| forward | Hc_bt1_F1_0N | TCGTCGGCAGCGTCAGATGTGTATAAGAGACAGACGCATTCACTTGGAGGAGG |
|  | Hc_bt1_F1_1N | TCGTCGGCAGCGTCAGATGTGTATAAGAGACAGNACGCATTCACTTGGAGGAGG |
|  | Hc_bt1_F1_2N | TCGTCGGCAGCGTCAGATGTGTATAAGAGACAGNNACGCATTCACTTGGAGGAGG |
|  | Hc_bt1_F1_3N | TCGTCGGCAGCGTCAGATGTGTATAAGAGACAGNNNACGCATTCACTTGGAGGAGG |
| forward | Hc_bt1_f2_0N | TCGTCGGCAGCGTCAGATGTGTATAAGAGACAGACGCATTCGCTTGGAGGAG |
|  | Hc_bt1_f2_1N | TCGTCGGCAGCGTCAGATGTGTATAAGAGACAGNACGCATTCGCTTGGAGGAG |
|  | Hc_bt1_f2_2N | TCGTCGGCAGCGTCAGATGTGTATAAGAGACAGNNACGCATTCGCTTGGAGGAG |
|  | Hc_bt1_f2_3N | TCGTCGGCAGCGTCAGATGTGTATAAGAGACAGNNNACGCATTCGCTTGGAGGAG |
| forward | COOP_bt1_f1_0N | TCGTCGGCAGCGTCAGATGTGTATAAGAGACAGACGCATTCTCTTGGAGGAGG |
|  | COOP_bt1_f1_1N | TCGTCGGCAGCGTCAGATGTGTATAAGAGACAGNACGCATTCTCTTGGAGGAGG |
|  | COOP_bt1_f1_2N | TCGTCGGCAGCGTCAGATGTGTATAAGAGACAGNNACGCATTCTCTTGGAGGAGG |
|  | COOP_bt1_f1_3N | TCGTCGGCAGCGTCAGATGTGTATAAGAGACAGNNNACGCATTCTCTTGGAGGAGG |
| forward | Oo_bt1_f2_0N | TCGTCGGCAGCGTCAGATGTGTATAAGAGACAGACGCACTCTTTGGGAGGAG |
|  | Oo_bt1_f2_1N | TCGTCGGCAGCGTCAGATGTGTATAAGAGACAGNACGCACTCTTTGGGAGGAG |
|  | Oo_bt1_f2_2N | TCGTCGGCAGCGTCAGATGTGTATAAGAGACAGNNACGCACTCTTTGGGAGGAG |
|  | Oo_bt1_f2_3N | TCGTCGGCAGCGTCAGATGTGTATAAGAGACAGNNNACGCACTCTTTGGGAGGAG |
| forward | Tcol_bt1_f3_0N | TCGTCGGCAGCGTCAGATGTGTATAAGAGACAGACACATTCTCTTGGAGGAGGTAC |
|  | Tcol_bt1_f3_1N | TCGTCGGCAGCGTCAGATGTGTATAAGAGACAGNACACATTCTCTTGGAGGAGGTAC |
|  | Tcol_bt1_f3_2N | TCGTCGGCAGCGTCAGATGTGTATAAGAGACAGNNACACATTCTCTTGGAGGAGGTAC |
|  | Tcol_bt1_f3_3N | TCGTCGGCAGCGTCAGATGTGTATAAGAGACAGNNNACACATTCTCTTGGAGGAGGTAC |
| forward | Tc_bt1_F1_0N | TCGTCGGCAGCGTCAGATGTGTATAAGAGACAGACGCATTCTTTGGGAGGAGG |
|  | Tc_bt1_F1_1N | TCGTCGGCAGCGTCAGATGTGTATAAGAGACAGNACGCATTCTTTGGGAGGAGG |
|  | Tc_bt1_F1_2N | TCGTCGGCAGCGTCAGATGTGTATAAGAGACAGNNACGCATTCTTTGGGAGGAGG |
|  | Tc_bt1_F1_3N | TCGTCGGCAGCGTCAGATGTGTATAAGAGACAGNNNACGCATTCTTTGGGAGGAGG |
| forward | Tc_bt1_f2_0N | TCGTCGGCAGCGTCAGATGTGTATAAGAGACAGACGCATTCCTTGGGAGGAG |
|  | Tc_bt1_f2_1N | TCGTCGGCAGCGTCAGATGTGTATAAGAGACAGNACGCATTCCTTGGGAGGAG |
|  | Tc_bt1_f2_2N | TCGTCGGCAGCGTCAGATGTGTATAAGAGACAGNNACGCATTCCTTGGGAGGAG |
|  | Tc_bt1_f2_3N | TCGTCGGCAGCGTCAGATGTGTATAAGAGACAGNNNACGCATTCCTTGGGAGGAG |
|  |  |  |
| reverse | Hc_bt1_R1_0N | GTCTCGTGGGCTCGGAGATGTGTATAAGAGACAGTGTGAGTTTCAAAGTGCGGAAG |
|  | Hc_bt1_R1_1N | GTCTCGTGGGCTCGGAGATGTGTATAAGAGACAGNTGTGAGTTTCAAAGTGCGGAAG |
|  | Hc_bt1_R1_2N | GTCTCGTGGGCTCGGAGATGTGTATAAGAGACAGNNTGTGAGTTTCAAAGTGCGGAAG |
|  | Hc_bt1_R1_3N | GTCTCGTGGGCTCGGAGATGTGTATAAGAGACAGNNNTGTGAGTTTCAAAGTGCGGAAG |
| reverse | Hc_bt1_R2_0N | GTCTCGTGGGCTCGGAGATGTGTATAAGAGACAGGCGAGTTTCAAAGTGCGGAAG |
|  | Hc_bt1_R2_1N | GTCTCGTGGGCTCGGAGATGTGTATAAGAGACAGNGCGAGTTTCAAAGTGCGGAAG |
|  | Hc_bt1_R2_2N | GTCTCGTGGGCTCGGAGATGTGTATAAGAGACAGNNGCGAGTTTCAAAGTGCGGAAG |
|  | Hc_bt1_R2_3N | GTCTCGTGGGCTCGGAGATGTGTATAAGAGACAGNNNGCGAGTTTCAAAGTGCGGAAG |
| reverse | Coop_bt1_r1_0N | GTCTCGTGGGCTCGGAGATGTGTATAAGAGACAGGTGAGCTTCAATGTGCGGAAG |
|  | Coop_bt1_r1_1N | GTCTCGTGGGCTCGGAGATGTGTATAAGAGACAGNGTGAGCTTCAATGTGCGGAAG |
|  | Coop_bt1_r1_2N | GTCTCGTGGGCTCGGAGATGTGTATAAGAGACAGNNGTGAGCTTCAATGTGCGGAAG |
|  | Coop_bt1_r1_3N | GTCTCGTGGGCTCGGAGATGTGTATAAGAGACAGNNNGTGAGCTTCAATGTGCGGAAG |
| reverse | Coop_bt1_R2_0N | GTCTCGTGGGCTCGGAGATGTGTATAAGAGACAGTGAGCTTCAAGGTGCGGAAG |
|  | Coop_bt1_R2_1N | GTCTCGTGGGCTCGGAGATGTGTATAAGAGACAGNTGAGCTTCAAGGTGCGGAAG |
|  | Coop_bt1_R2_2N | GTCTCGTGGGCTCGGAGATGTGTATAAGAGACAGNNTGAGCTTCAAGGTGCGGAAG |
|  | Coop_bt1_R2_3N | GTCTCGTGGGCTCGGAGATGTGTATAAGAGACAGNNNTGAGCTTCAAGGTGCGGAAG |
| reverse | Coop_bt1_R3_0N | GTCTCGTGGGCTCGGAGATGTGTATAAGAGACAGGAGCTTCAACGTGCGGAAG |
|  | Coop_bt1_R3_1N | GTCTCGTGGGCTCGGAGATGTGTATAAGAGACAGNGAGCTTCAACGTGCGGAAG |
|  | Coop_bt1_R3_2N | GTCTCGTGGGCTCGGAGATGTGTATAAGAGACAGNNGAGCTTCAACGTGCGGAAG |
|  | Coop_bt1_R3_3N | GTCTCGTGGGCTCGGAGATGTGTATAAGAGACAGNNNGAGCTTCAACGTGCGGAAG |
| reverse | Oo_bt1_r2_0N | GTCTCGTGGGCTCGGAGATGTGTATAAGAGACAGTGTGAGTTTTAGTGTGCGGAAG |
|  | Oo_bt1_r2_1N | GTCTCGTGGGCTCGGAGATGTGTATAAGAGACAGNTGTGAGTTTTAGTGTGCGGAAG |
|  | Oo_bt1_r2_2N | GTCTCGTGGGCTCGGAGATGTGTATAAGAGACAGNNTGTGAGTTTTAGTGTGCGGAAG |
|  | Oo_bt1_r2_3N | GTCTCGTGGGCTCGGAGATGTGTATAAGAGACAGNNNTGTGAGTTTTAGTGTGCGGAAG |
| reverse | Tcol_bt1_R2_0N | GTCTCGTGGGCTCGGAGATGTGTATAAGAGACAGGTGAGCTTCAATGTGCGGAAA |
|  | Tcol_bt1_R2_1N | GTCTCGTGGGCTCGGAGATGTGTATAAGAGACAGNGTGAGCTTCAATGTGCGGAAA |
|  | Tcol_bt1_R2_2N | GTCTCGTGGGCTCGGAGATGTGTATAAGAGACAGNNGTGAGCTTCAATGTGCGGAAA |
|  | Tcol_bt1_R2_3N | GTCTCGTGGGCTCGGAGATGTGTATAAGAGACAGNNNGTGAGCTTCAATGTGCGGAAA |
| reverse | Tcol_bt1_R3_0N | GTCTCGTGGGCTCGGAGATGTGTATAAGAGACAGTGTAAGCTTAAGTGTTCGGAAGC |
|  | Tcol_bt1_R3_1N | GTCTCGTGGGCTCGGAGATGTGTATAAGAGACAGNTGTAAGCTTAAGTGTTCGGAAGC |
|  | Tcol_bt1_R3_2N | GTCTCGTGGGCTCGGAGATGTGTATAAGAGACAGNNTGTAAGCTTAAGTGTTCGGAAGC |
|  | Tcol_bt1_R3_3N | GTCTCGTGGGCTCGGAGATGTGTATAAGAGACAGNNNTGTAAGCTTAAGTGTTCGGAAGC |
| reverse | Tc_bt1_R1_0N | GTCTCGTGGGCTCGGAGATGTGTATAAGAGACAGTGTGAGTTTTAAGGTGCGGAAG |
|  | Tc_bt1_R1_1N | GTCTCGTGGGCTCGGAGATGTGTATAAGAGACAGNTGTGAGTTTTAAGGTGCGGAAG |
|  | Tc_bt1_R1_2N | GTCTCGTGGGCTCGGAGATGTGTATAAGAGACAGNNTGTGAGTTTTAAGGTGCGGAAG |
|  | Tc_bt1_R1_3N | GTCTCGTGGGCTCGGAGATGTGTATAAGAGACAGNNNTGTGAGTTTTAAGGTGCGGAAG |
| reverse | Tc_bt1_R2_0N | GTCTCGTGGGCTCGGAGATGTGTATAAGAGACAGTGGGAGTTTTAAGGTGCGGAAG |
|  | Tc_bt1_R2_1N | GTCTCGTGGGCTCGGAGATGTGTATAAGAGACAGNTGGGAGTTTTAAGGTGCGGAAG |
|  | Tc_bt1_R2_2N | GTCTCGTGGGCTCGGAGATGTGTATAAGAGACAGNNTGGGAGTTTTAAGGTGCGGAAG |
|  | Tc_bt1_R2_3N | GTCTCGTGGGCTCGGAGATGTGTATAAGAGACAGNNNTGGGAGTTTTAAGGTGCGGAAG |
| reverse | Tc_bt1_R3_0N | GTCTCGTGGGCTCGGAGATGTGTATAAGAGACAGGTGAGTTTCAAGGTGCGGAAG |
|  | Tc_bt1_R3_1N | GTCTCGTGGGCTCGGAGATGTGTATAAGAGACAGNGTGAGTTTCAAGGTGCGGAAG |
|  | Tc_bt1_R3_2N | GTCTCGTGGGCTCGGAGATGTGTATAAGAGACAGNNGTGAGTTTCAAGGTGCGGAAG |
|  | Tc_bt1_R3_3N | GTCTCGTGGGCTCGGAGATGTGTATAAGAGACAGNNNGTGAGTTTCAAGGTGCGGAAG |

**Supplementary Table 2.** Prevalence of helminths and protozoan parasites from Fecal Egg Counts by state for commercial bison herds.

| Herd (n) | Parasite | No. of positive herds | Prevalence  (%) |
| --- | --- | --- | --- |
| Texas (n = 14) | *Strongyle* | 14 | 100 |
|  | *Eimeria* | 13 | 92.9 |
|  | *Trichuris* | 7 | 50.0 |
|  | *Moniezia* | 10 | 71.4 |
|  | *Strongyloides* | 3 | 21.4 |
|  | *Capillaria* | 2 | 14.3 |
|  | *Nematodirus* | 2 | 14.3 |
| Oklahoma (n =2) | *Strongyle* | 2 | 100 |
|  | *Eimeria* | 2 | 100 |
|  | *Trichuris* | 2 | 100 |
|  | *Moniezia* | 2 | 100 |
|  | *Strongyloides* | 1 | 50.0 |
|  | *Capillaria* | 1 | 50.0 |
|  | *Nematodirus* | 2 | 100 |

**Supplementary Table 3.** Prevalence of helminths and protozoan parasites from Fecal Egg Counts by age class from a single commercial bison herd in east-central Texas

| Herd (n) | Parasite | No. of positive herds | Prevalence  (%) |
| --- | --- | --- | --- |
| Calves (n = 14) | *Strongyle* | 14 | 100 |
|  | *Eimeria* | 11 | 78.6 |
|  | *Trichuris* | 5 | 35.7 |
|  | *Moniezia* | 3 | 21.4 |
| Yearling (n =3) | *Strongyle* | 3 | 100 |
|  | *Eimeria* | 3 | 100 |
|  | *Trichuris* | 1 | 33.3 |
|  | *Moniezia* | 0 | n/a |
| Mature (n =26) | *Strongyle* | 16 | 61.5 |
|  | *Eimeria* | 7 | 26.9 |
|  | *Trichuris* | 1 | 3.8 |
|  | *Moniezia* | 2 | 7.7 |
